# Reparation of an Inflamed Air-Liquid Interface Cultured A549 Cells with Nebulized Nanocurcumin

**DOI:** 10.3390/pharmaceutics13091331

**Published:** 2021-08-25

**Authors:** Maria Julia Altube, Lilen Ivonne Caimi, Cristian Huck-Iriart, Maria Jose Morilla, Eder Lilia Romero

**Affiliations:** 1 Nanomedicines Research and Development Centre (NARD), Science and Technology Department, National University of Quilmes, Roque Sáenz Peña 352, Bernal B1876, Argentina; julia.altube@unq.edu.ar (M.J.A.); jmorilla@unq.edu.ar (M.J.M.); 2 Cell Cycle and Genomic Stability Laboratory, Fundación Instituto Leloir, Av. Patricias Argentinas 435, Buenos Aires C1405, Argentina; lilencaimi@gmail.com; 3 Applied Crystallography Laboratory, Science and Technology School, National University of San Martin (UNSAM)-CONICET, San Martín B1650, Argentina; chuck@unsam.edu.ar

**Keywords:** curcumin, nanovesicles, archaeolipids, A549 cells, lung injury, proinflammatory cytokines

## Abstract

The anti-inflammatory, antifibrotic and antimicrobial activities of curcumin (CUR) are missed because of its low solubility in aqueous media, low bioavailability, and structural lability upon oral intake. Soft nanoparticles such as nanoliposomes are not efficient as CUR carriers, since crystalline CUR is expelled from them to physiological media. Nanostructures to efficiently trap and increase the aqueous solubility of CUR are needed to improve both oral or nebulized delivery of CUR. Here we showed that SRA1 targeted nanoarchaeosomes (nATC) [1:0.4 w:w:0.04] archaeolipids, tween 80 and CUR, 155 ± 16 nm sized of −20.7 ± 3.3 z potential, retained 0.22 mg CUR ± 0.09 per 12.9 mg lipids ± 4.0 (~600 μM CUR) in front to dilution, storage, and nebulization. Raman and fluorescence spectra and SAXS patterns were compatible with a mixture of enol and keto CUR tautomers trapped within the depths of nATC bilayer. Between 20 and 5 µg CUR/mL, nATC was endocytosed by THP1 and A549 liquid–liquid monolayers without noticeable cytotoxicity. Five micrograms of CUR/mL nATC nebulized on an inflamed air–liquid interface of A549 cells increased TEER, normalized the permeation of LY, and decreased il6, tnfα, and il8 levels. Overall, these results suggest the modified pharmacodynamics of CUR in nATC is useful for epithelia repair upon inflammatory damage, deserving further deeper exploration, particularly related to its targeting ability.

## 1. Introduction

Respiratory diseases causing severe lung damage characterised by epithelial injury, airway inflammation, defective tissue repair, and airway remodelling, are among the leading causes of death worldwide [1]. The recovery after long-term inflammation or acute injury, caused by pathogen challenge or acute respiratory distress syndrome (ARDS) [2] may be mediated by abnormal wound healing, and end up in the formation of scar tissue or fibrosis [3]. Pulmonary fibrosis is a progressive disease characterized by a widespread accumulation of myofibroblasts and extracellular matrix components. The infection with severe acute respiratory syndrome coronavirus 2 (SARS-CoV-2), the causing agent of COVID-19 for instance, causes diffuse alveolar damage (DAD), and further fibrotic remodelling with persistent reduced oxygenation. The global burden of fibrotic lung disease following SARS-CoV-2 infection is predicted to be high in the following months and years [4].

Anti-inflammatory agents alone, are not sufficient to avoid the establishment of fibrotic changes [5]. Pirfenidone and nintedanib are the only two FDA approved drugs against liver fibrosis and idiopathic pulmonary fibrosis (IPF), currently under test to prevent or impair the worsening of post-COVID fibrosis [4,6]. Both drugs are orally administered and cause undesirable systemic side effects [7,8].

Inhalation on the other hand, is the administration route that provides a direct access of drugs to lungs epithelia. However, the market of inhaled agents is mastered by anti-inflammatory drugs, namely corticosteroids, leukotriene antagonists, and mast cell stabilizers, used to treat chronic oobstructive pulmonary disease (COPD), asthma, bronchitis, mesothelioma, lung cancer, or allergies [9]. A single preclinical report was recently published on the performance of an inhaled antifibrotic agent [10]. Moreover, the inhalation of nanoparticulate drug formulations instead of free drugs, allows to adjust site specific biodistribution (by impairing drug’s systemic access, while targeting them to the large surface of the lung epithelia), with tailored pharmacokinetics (enlarging their residence time on low metabolic activity epithelia) and pharmacodynamics (by targeting to selected cells with lower dosage, minimizing adverse reactions) [11,12,13]. Currently, the antimicrobial liposomal amikacin Arikayce is the only inhaled nanoparticle currently on the market [14].

Here we present the structural features and in vitro performance of nebulized nanoarchaeosomes loaded with CUR, on inflamed alveolar type II epithelial cells models. CUR is a hydrophobic polyphenol extracted from turmeric with well-known effects on multiple targets, that besides of antioxidant, antitumoral, and antimicrobial, also displays anti-inflammatory and antifibrotic activities [15,16]. Nanoarchaeosomes are nanovesicles made of *sn*1,2 glycerol ether polar archaeolipids, with fully saturated polyisoprenoid chains [17]. The archaeolipids used here were extracted from the halophilic archaebacteria *Halorubrum tebenquichense* and ordered in decrescent abundance [18] are: archaeol analog methyl ester of phosphatidylglycerophosphate (PGPMe), archaeol analog phosphatidylglycerol (PG), (1-O-[α-D-mannose-(2′-SO3H)-(1′ α 2′)-α-D-glucose]-2,3-di-O-phytanyl-sn-glycerol) (SDGD5) the cardiolipin bis phosphatidylglycerol (BPG) and the glycocardiolipin SDGD5PA (2′-SO3H)-Manp-α1,2Glcpα-1-1-[sn-2,3-di-Ophytanylglycerol]-6-[phospho-sn-2,3-di-O-phytanylglycerol].

Nanoarchaeosomes display higher structural resistance to enzymatic, physicochemical, and mechanical stress than nanoliposomes prepared with lipids extracted from plants, animals, or bacteria [19,20]. Different from liposomes, the nanoarchaeosomes prepared with archaeolipids from *H. tebenquichense* are naturally targeted to cells expressing SRA1 [21] such as Kupffer cells, splenic, thymic, and alveolar macrophages [22,23,24], endothelial cells lining the liver and adrenal sinusoids [22,23] and on the high endothelial cells of postcapillary venules in the lymph nodes [25].

In this preliminary work, we hypothesized that the unique chemical structure of archaeolipids would be of aid to efficiently trap poorly soluble drugs such as CUR. Counting on CUR loaded nanovesicles refractory to mechanical and chemoenzymatic insults, biocompatible and importantly, capable of avoiding CUR release when diluted in aqueous media, is the first step to make profit of targeted CUR.

The chronic injury to distal lung tissue leads to either loss or altered function of alveolar type II cells, acknowledged as epithelial stem cells, that promote dysregulated repair and pathogenic activation of fibroblasts with progressive loss of lung function [5,26,27]. Inhaled medication capable of inducing alveolar epithelial repair, hence, may be more efficient than oral medication to impair the advance of fibrotic changes Here we found that besides of displaying anti-inflammatory activity, nebulized CUR loaded nanoarchaeosomes rebuilt the barrier properties of an air–liquid interface (ALI) in vitro model made of A549 cells, potentially constituting novel lung epithelial repairing agents.

## 2. Materials and Methods

### 2.1. Materials

1,2-Hydrogenated-L-α-phosphatidylcholine (HSPC) was from Northern Lipids Inc, Vancouver, Canadá. CUR, cholesterol (Chol), Tween 80, (6-Dodecanoyl-*N*,*N*-dimethyl-2-naphthylamine (Laurdan), 3-(4,5-dimetiltiazol-2-yl)-2,5-diphenyl tetrazolium bromide (MTT), phorbol 12-13-acetate (PMA), Mowiol, 2-mercaptoethanol, lipopolysaccharide (LPS) and dexamethasone 21-phosphate disodium salt (DEX) were from Sigma-Aldrich (MO, USA). Hoechst 33342, CellMask Deep Red Plasma membrane stain, 5-(and-6)-chloromethyl-2′,7′-dichlorodihydrofluorescein diacetate, acetyl ester (carboxy-H2DCFDA), Lucifer Yellow (LY), Roswell Park Memorial Institute 1640 (RPMI), Modified Eagle Medium (MEM), penicillin-streptomycin sulphate, glutamine, sodium pyruvate and trypsin/ethylenediamine tetra acetic acid were from Life Technologies (NY, USA), fetal bovine serum (FBS) was from Internegocios (Buenos Aires, Argentina). The other reagents were of analytic grade from Anedra, Research AG (Buenos Aires, Argentina).

### 2.2. Archaebacteria Growth, Extraction, and Characterization of Total Polar Archaeolipids (TPA)

*H. tebenquichense* c in basal medium supplemented with yeast extract and glucose [28]. Biomass was grown in 16.5-L medium in a 25-L homemade stainless-steel bioreactor at 40 °C and harvested 72 h after growth. TPA were extracted from biomass using the Bligh and Dyer method modified for extreme halophiles [29]. Around 700 mg TPA were isolated from each culture batch. The reproducibility of each TPA-extract composition was routinely screened by phosphate content [30] and electrospray-ionization mass spectrometry [18].

### 2.3. Preparation of CUR-Nanovesicles

Ordinary nanoliposomes (HSPC:Chol 1:0.33 w:w, nL), nL loaded with CUR (HSPC:Chol: CUR at 1:0.33: 0.053 w:w, nLC), nL containing the detergent and border activator Tween 80 (HSPC:Chol:Tween80 1:0.33:0.53 w:w, nLT), nLT loaded with CUR (HSPC:Chol:Tween80:CUR at 1:0.33:0.53:0.053 w:w, nLTC), nanoarchaeosomes (TPA 100%, nA), and nA loaded with CUR (TPA: CUR at 1: 0.04 w:w, nAC), nA containing Tween 80 (TPA:Tween80, 1:0.4 w:w, nAT) and nAT loaded with CUR (TPA:Tween80:CUR at 1:0.4:0.04 w:w, nATC) were prepared by the film hydration method. Briefly, mixtures of lipids and CUR dissolved in chloroform: methanol 1:1 *v/v* were mixed in round bottom flasks and were rotary evaporated at 40 °C until elimination of the solvent. The film was flushed with N_2_ and hydrated with aqueous phase (10 mM Tris buffer pH 7.4 with 0.9% w:w, NaCl-Tris buffer) up to a final concentration of 25 mg/mL of total lipids, 10 mg/mL of T80 and 1 mg/mL of CUR. The resultant suspensions were sonicated (1 h with a bath-type sonicator 80 W, 80 kHz). Samples were centrifuged at 5000× *g* for 30 s and CUR pellet was discarded to remove free CUR. All samples were extruded five times through two stacked polycarbonate membranes of 0.4 and 0.2 μm pore size using a 100 mL Thermobarrel extruder (Northern Lipids, BC, Canada). The resulting nanovesicles were filtered through a 0.22 μm sterile nylon membrane to sterilize the samples and for removing free CUR. Samples were stored at 4 °C protected from light.

### 2.4. Structural Characterization of CUR-Nanovesicles

Phospholipids were quantified by a colorimetric phosphate microassay [30].

CUR was quantified by absorbance at 425 nm upon complete disruption of one volume of nanovesicles suspension in 75 volumes of methanol. The absorption of the sample was compared to a standard curve prepared in methanol from a stock solution of 1 mg/mL of CUR in chloroform: methanol 1:1 *v*/*v*. The standard curve was linear in the range 0.125–4 µg/mL CUR, with correlation coefficient of 0.999. CUR calibration curve is provided in Supplementary Files.

Size and ζ potential were determined by dynamic light scattering and phase analysis light scattering, respectively, using a nanoZsizer apparatus (Malvern Instruments, Malvern, UK).

Cryo-electron microscopy (Cryo-EM) was performed at National Laboratory of Nanotechnology (LNNano) at CNPEM-Campinas, São Paulo. A 300 mesh Holey Lacey Carbon grid from Ted Pella^®^ was used, the grids were submitted to a glow discharge procedure (Pelco easi Glow discharge system Ted Pella, Redding, CA, USA) (15 mA for 10 s) in order to increase its hydrophilicity. Then grids were inserted in a Vitrobot^®^ (Mark IV, Thermo Fischer Scientific, Waltham, MA, USA) where 3 µL of sample were added, it was given 20 s for sample fixation. Subsequently, an automatic blotting (blot force = −5, blot total = 3) was performed to dry the excess of sample. Finally, the grid was rapidly plunged into liquid ethane wrapped into a liquid nitrogen environment. Measurements were made in a TALOS F200C (Thermo Fischer Scientific, Waltham, MA, USA) microscope at 200 kV with a CMOS camera Ceta 16M 4K × 4K (Thermo Fischer Scientific, Waltham, MA, USA). ImageJ^®^ software (National Institutes of Health, 1987) was used to image manipulation.

The order and fluidity of the nanovesicles bilayer with or without CUR were assessed by determining the Laurdan generalized polarization (GP) and fluorescence anisotropy (FA) in the nanovesicles. Nanovesicles were labelled with Laurdan by mixing 10 µL of 120 mM Laurdan in methanol with a volume of nanovesicles sufficient to render a 1: 20 mol: mol Laurdan: lipid ratio. GP was calculated using the following equation:

$$\mathrm{GP}=\frac{I_{440}-I_{490}}{I_{440}+I_{490}}$$

where *I*_440_ and *I*_490_ are the fluorescence intensities at λem = 440 nm and λem = 490 nm, respectively, obtained from the spectra between 400 and 520 nm at λex =364 nm (slit ex: 5.0 nm and slit em: 10.0 nm. Scan speed: 100 nm min^−1^). FA was calculated using the fluorimeter software according to the following equation:

$$\mathrm{FA}=\frac{I_{0}-GI_{90}}{I_{0}+2GI_{90}}$$

where *I*_0_ and *I*_90_ are the fluorescence intensities at λem = 440 nm with λex = 364 nm, and the excitation polarizer is oriented at 0 and 90° respectively. The correction factor G was obtained from the ratio of emission intensity at 0 and 90°, with the excitation polarizer oriented at 90° (after the subtraction of scattered light).

#### 2.4.1. Raman Spectra

Raman measurements were obtained with a i-Raman BWS465-785S BWTek spectrometer equipped with Video Microscope (BAC151B model) at the Instituto de Investigaciones Fisicoquímicas Teoricas y Aplicadas (INIFTA, Buenos Aires, Argentina). The excitation wavelength was 785 nm and the laser was focused on the sample using a 20× optical magnification. Spectra were recorded in the region of 600 to 2000 cm^−1^.

#### 2.4.2. Small Angle X-ray Scattering (SAXS)

SAXS measurements were performed using XEUSS 1.0 (from XENOCS, France) equipment at INIFTA facilities. Two dimensional patterns were registered with a 2D photon counting pixel X-ray detectors for SAXS Pilatus 100 k (DECTRIS, Switzerland). SAXS measurements were done at a 1300 mm sample to detector distance. In addition, 1D scattering intensity, I(q), was obtained through integration in the range of the momentum transfer q, where q = 4π⁄λ sin(θ), 2θ is the scattering angle and λ = 0.15419 nm is the weighted average of X-ray wavelength of the Cu-K_α12_ emission lines. Due to the small beam size at the sample (500 × 500 μm^2^) smearing effects were not taken into account. Solutions were placed at room temperature using in-house sample holders for liquids with kapton^®^ windows. All measurements were done in transmission mode. Patterns were taken for 90 min each. SAXS model description is provided in Supplementary Files [31,32,33,34,35,36].

#### 2.4.3. Absorption Spectra of Free CUR in NaCl-Tris Buffer or Methanol and CUR-Nanovesicles

The spectra were carried out at 2 µg/mL of CUR using a spectrophotometer UV-9000S Shanghai Yoke Instrument Co., Ltd., Shanghai, China.

Fluorescence emission measurements of CUR in methanol and CUR-nanovesicles at 2 µg/mL CUR (λex = 425 nm) were obtained with a fluorescence spectrometer LS Perkin Elmer.

#### 2.4.4. Stability upon Storage

The colloidal stability of nanovesicles was determined after 1-, 2-, and 6-months storage at 4 °C. Nanovesicles size and Z potential before and after storage were determined as stated before. The presence of CUR crystals was determined by color brightfield microscopy. For this purpose, nATC and nLTC were diluted with RPMI medium in a 24 wells plate at a final concentration of 20 µg/mL of CUR. Dilutions were observed with 20× magnification using Cytation™ 5 (BioTek Instruments, Inc., Winooski, VT, USA).

#### 2.4.5. Stability of CUR-Nanovesicles and Free CUR upon Nebulization

The structural stability upon nebulization of freshly prepared nanovesicles and after 6 months storage at 4 °C was determined in terms of size, polydispersity index, total lipids, and CUR recovery. To compare the nebulization performance of CUR-nanovesicles and free CUR, CUR powder was dissolved in dimethyl sulfoxide (DMSO) to make a 5 mg/mL stock solution and diluted with buffer Tris pH 7.4 0.9% *m*/*v* at a concentration of 30 μg/mL. Briefly, 2 mL of CUR-nanovesicles or free CUR were nebulized in a vibrating mesh nebulizer (Omron NE-U22, OMRON Healthcare, Kyoto, Japan) for 5 min. The aerosols were collected in a vessel connected to the nebulizer. Nanovesicles size, total lipids, and CUR were quantified as stated above, before and after nebulization.

### 2.5. Cell Lines and Culture

Human epithelial lung cell line A549 (ATCC^®^ CCL-185™) and human rhabdomyosarcoma cell line RD (ATCC^®^ CCL-136™) were maintained in MEM. Mediums were supplemented with 10% FBS, 100 U/mL penicillin, 100 μg/mL streptomycin and 2 mM L-glutamine. The human monocyte cell line THP-1 (ATCC TIB- 202™) were maintained in RMPI medium supplemented with 100 U/mL penicillin, 100 μg/mL streptomycin and 2 mM L-glutamine, 0.05 mM 2-mercaptoethanol and 1 mM sodium pyruvate. THP-1, monocytes, were differentiated into macrophages by treatment with 100 ng/mL PMA for 24 h. All cell lines were grown in a humidified atmosphere of 5% CO_2_ at 37 °C.

### 2.6. Cytotoxicity of CUR-Nanovesicles

The viability of cells upon 24 h incubation with nanovesicles was measured by the MTT assay. Briefly, THP-1, A549 and RD cells were seeded in 96-well plates with a density of 4 × 10^4^ cells per well and grown for 24 h. Then, cells were incubated with a series of different concentration of empty nanovesicles (350, 700, and 1400 µg/mL of total lipids), free CUR (CUR) and CUR-nanovesicles (5, 10, and 20 μg/mL of CUR). After 24 h of incubation the medium was removed, cells were washed with PBS and 100 μL of 5 mg/mL MTT solution was added to each well. After 3 h of incubation, the MTT solution was removed, the insoluble formazan crystals were dissolved in dimethyl sulfoxide, and absorbance was measured at 570 nm. The cell viability was expressed as a percentage of the cells grown in medium.

### 2.7. Lactate Dehydrogenase (LDH) Leakage

THP-1 cells were seeded in 96-well plates with a density of 4 × 10^4^ cells per well and grown for 24 h. Then, cells were incubated with series of different concentration of empty nanovesicles (350, 700, and 1400 µg/mL of total lipids), CUR and CUR-nanovesicles (5, 10, and 20 μg/mL of CUR) in the absence or presence of LPS (to screen for cytotoxicity in an activated macrophage model, usually presented in inflammation). After 24 h, the supernatants were transferred to a fresh plate, centrifuged at 250× *g* for 5 min, and the LDH leakage in culture supernatants was measured using a CytoTox LDH kit (Promega, Madison, WI, USA). Percent of LDH released was expressed relative to treatment with lysis solution.

### 2.8. CUR Cellular Uptake

THP-1 cells were seeded in 96-well plates with a density of 4 × 10^4^ cells per well and grown for 24 h. Then, cells were incubated with series of different concentration of empty nanovesicles (350, 700, and 1400 µg/mL of total lipids), CUR and CUR-nanovesicles (5, 10, and 20 μg/mL of CUR). After 24 h, cells were washed three times with PBS and CUR fluorescence intensity of whole cells was determined in each well at λex = 425 nm and λem = 490 nm. In addition, cells were observed at bright field with 20× magnification using Cytation™ 5 (BioTek Instruments, Inc., Winooski, VT, USA).

### 2.9. Inhibition of Reactive Oxygen Species (ROS)

The capacity of nanovesicles to reduce the generation of ROS stimulated with LPS, was measured using the carboxy-H2DCFDA dye. Differentiated THP-1 cells were seeded at a density of 4 × 10^4^ cells per well onto 96-well plates and grown for 24 h at 37 °C. Then, cells incubated with fresh medium with 5% FBS containing void nanovesicles (nLT and nAT at 350 µg total lipids/mL) or CUR-nanovesicles (nLTC and nATC, at 5 µg CUR/mL and 350 µg total lipids/mL) and CUR (5 µg CUR/mL) with and without 1 µg/mL of LPS. After 24 h incubation, cells attached at the bottom of the well were washed twice with PBS, incubated with a solution of 10 µM carboxy-H2DCFDA for 30 min at 37 °C. Then, cells were washed with PBS and fluorescence intensity of whole cells was measured using Cytation™ 5 (BioTek Instruments, Inc., Winooski, VT, USA) at λex = 490 nm and λem = 520 nm.

### 2.10. Cytokine Release

The in vitro anti-inflammatory activity of nanovesicles was determined by measuring the release of pro-inflammatory cytokines by cells stimulated with LPS. Briefly, A549 and differentiated THP-1 cells were seeded at a density of 2 × 10^5^ cells per well into 24-well plates and grown for 24 h at 37 °C. Then cells were co-incubated with 1 µg/mL LPS and empty nanovesicles (nLT and nAT at 350 µg total lipids/mL), CUR-nanovesicles (nLTC and nATC at 5 µg CUR/mL and 350 µg total lipids/mL), CUR (5 µg/mL of CUR), or DEX (10 µg/mL). LPS-stimulated and non-stimulated cells without treatments were used as positive and negative controls, respectively. After 24 h of incubation, supernatants were collected and stored at −20 °C until analysis. Human TNF-α, IL-8, and IL-6 levels were measured by enzyme linked immunosorbent assay (BD OptEIA™, BD Biosciences, San Jose, CA, USA) following the manufacturer instructions. Absorbance measurements were carried out at 450 nm on a microplate reader in a microplate reader.

### 2.11. Anti-Inflammatory Activity of Nebulized CUR-Nanovesicles on an Inflamed Alveolar Epithelium Model at the Air Liquid Interface (ALI)

A549 cells were seeded in Greiner ThinCert™ multiwell plate insert (0.6 × 10^6^ cm^−2^ pore density PET membranes with a growth area of 113.1 mm^2^, 3.0 μm pores, and transparent optical membrane properties; Greiner Bio-One, Switzerland) placed in 12-well tissue culture plates at a density of 0.1 × 10^6^ cells/mL per insert. Cells were grown in MEM with 10% FBS at humidified atmosphere of 5% CO_2_ at 37 °C. After 24 h incubation the medium was replaced with polarization medium (MEM with 5% FBS and 200 nM DEX). After 48 h cells were transferred from submerged to ALI conditions. For this purpose, the medium from the apical side was removed and the medium in the basal side was replaced by 1200 µL of fresh polarization medium. After additional 24 h in the incubator at ALI, an inflammatory environment was created by adding 1 μg/mL (LPS) into the medium of the basal side. Suspensions of nATC and nLTC (5 µg/mL de CUR) were nebulized during 2 min using Omron NE-U22 nebulizer above cells monolayers. During nebulization, each insert was placed in a new plate to avoid cross-contamination between samples. The whole procedure was carried out in a biosafety cabinet and the laminar flow was turned down during the nebulization. After that, each insert was placed again in the chamber with medium with or without LPS. DEX (10 µg/mL) was used as a positive control. The cells were then incubated at 37 °C in 5% CO_2_ humidified atmosphere.

IL-8 and IL-6 release was determined after 24 h of nanovesicles nebulization in the medium of the basal side.

Transepithelial electric resistance (TEER) was measured with an epithelial voltohmeter (Millicell-ERS-electrode, MERSS00001, Millipore Millicell; Billerica, MA, USA). The mean of three measurements per insert was determined. Before each measurement the cells monolayers were allowed to equilibrate for 15 min at room temperature in the biological safety cabinet. The electrical resistance of inserts without cells was subtracted from all samples, and the resistance values were multiplied with the surface area of the inserts (1.131 cm^2^).

The permeability of A549 cell monolayer was determined by measuring the passive transport of LY molecules across the monolayer. After 24 h post nebulization the medium at the basal side was replaced by 1500 µL of PBS with 5% FBS. At the apical side of the cell monolayer 500 μL of 100 µg/mL LY solution in PBS was added. LY in equilibrium was determined in an insert without cells. The cells were then incubated at 37 °C in 5% CO_2_ humidified atmosphere for two hours. Afterwards, the basal and apical medium was collected and the fluorescence intensity of LY was measured at λEx = 428 nm and λEm = 536 nm using a Cytation™ 5.

Confocal microscopy was performed to study cell monolayer integrity. After 24 h post nebulization cell monolayers were washed with PBS and incubated with 250 µL of CellMask™ Deep Red plasma membrane stain for 5 min at 37 °C. Then, cells were washed with PBS and stained with Hoechst 33,342 for 10 min at 37 °C. Afterwards cells were washed and fixed for 5 min with 3.75% formaldehyde in PBS. Finally, preparations were washed three times in PBS and mounted in Mowiol. A Leica laser-scanning spectral Confocal microscope TCS SP8; (Leica Microsystem, Wetzlar, Germany) was used. Image processing was performed using Fiji Software [37].

Statistical Analysis. Statistical analyses were performed by one-way analysis of variance followed by Dunnet’s test using Prisma 8.0 software (GraphPad, CA, USA). The *p*-value of <0.05 was considered statistically significant: * *p* < 0.05; ** *p* < 0.01; *** *p* < 0.001, **** *p* < 0.0001; n.s. represents nonsignificant (*p* > 0.05).

## 3. Results

### Physicochemical Characterization of CUR-Nanovesicles

In Table 1, the structural features of CUR-loaded nanoarchaeosomes (nAC) and Tween 80-containing nanoarchaeosomes (nATC), HSPC:Chol nanoliposomes (nLC) and Tween 80-containing HSPC:Chol nanoliposomes (nLTC), are shown. In Figure S3 representative images of CUR-nanovesicles suspensions are depicted.

Freshly prepared nAC contained ~80 µg CUR/mL (200 μM CUR, a concentration comparable to 190 μM CUR in 2.8 mM PC liposomes reported in [38]). CUR solubility as nATC was increased to 600 μM (threefold higher than in nAC and matching that reported in [39]), suggesting the presence of Tween 80, favoured the partition of CUR into bilayers. Overall, CUR’s solubility as nATC was increased by 417 folds (and by 300 folds as nLTC) compared to that in buffer tris pH 7.4 NaCl 0.9%.

**Figure 1:**
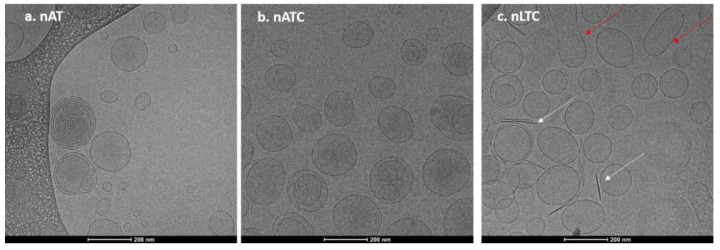
Representative Cryo-EM images of nanovesicles (**a**) nATC, (**b**) nAT, and (**c**) nLTC. White arrows point out CUR crystals and the red ones fused nanovesicles.

Nanovesicles morphology was determined by Cryo-EM. In Figure 1a,b, representative images of nAT (30–230 nm diameter nanovesicles) and of nATC (60–200 nm diameter nanovesicles) are shown. In Figure 1c, arrows point out 8 nm width and 140 nm mean length rods, probably CUR crystals expulsed from nLTC (90–200 nm nanovesicles) lipid bilayers. All nanovesicles were mostly unilamellar.

**Figure 2:**
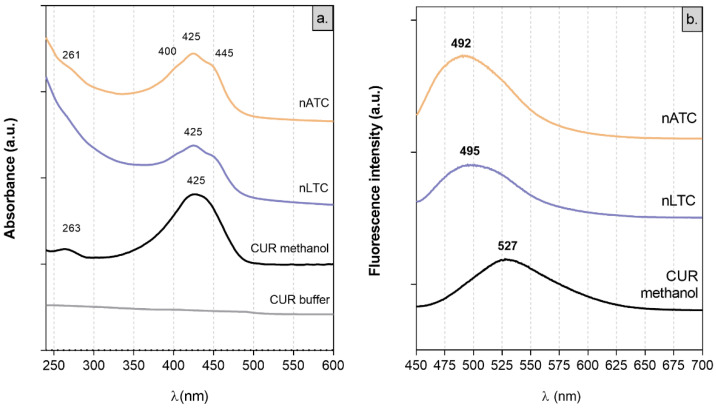
(**a**) absorption and (**b**) emission spectra of CUR alone or within nanovesicles.

Absorption spectra: Because of its low solubility, CUR does not present absorption peaks between 250 and 600 nm in buffer tris pH 7.4 [40]. In polar solvents, CUR absorbs visible light because of its conjugated double bonds. In solution, CUR displays keto-enol equilibrium, between a planar enol form and a less polar, more energetic, and angular diketo tautomer, where the conjugation between aromatic rings is interrupted [41]. The λmax absorption at 425 nm of CUR in methanol is ascribed to the enol-monoanion form; the two shoulders at 445 nm and 400 nm observed in nATC and nLTC suspensions are ascribed to the enol and diketo forms, respectively (Figure 2a) [38,40,42].

Fluorescence spectra: the displacement of the tautomeric equilibria of CUR depends on polarity and aprotic or protic character of the solvent. This is better observed in fluorescence but not in absorbance spectra [43]. The most the solvent perturbates the intramolecular cis-enol hydrogen bond (that limits CUR’s out of plane vibration), the higher is the bathochromic shift of CUR’s λmax emission. CUR’s λmax emission at 527 nm in methanol is shifted to 495 nm in nLTC and to 492 nm in nATC. Such a hypsochromic shift indicated that in nanovesicles, CUR is immersed in a highly non-polar environment (Figure 2b) [38,43].

Raman spectra: Raman spectra are useful to detect the intense olefinic stretching bands of the hydrocarbon chains as well as deformations of aromatic rings. Most of the Raman bands assignments of CUR in solid state have been published by [44]; briefly: (a) 1626 cm^−1^ (stretching vibrations ν (C=O) and ν (C=C) of the interring chain [IRC] (b) 1601 cm^−1^ (stretching vibration ν (C=C) of the aromatic ring [AR]; (c) 1431 cm^−1^ (in plane bending δ (CCC) and δ COH of AR [45]; (d) 1321 cm^−1^ (δ (C=C–H) of the IRC); (e) 1256 cm^−1^ (δ(C–H) of AR, associated to ν (C-O) of the ether groups linked to these rings [46]; (f) a strong band at 1184 cm^−1^ (ring skeletal deformations coupled to the phenolic ν (C–O)); (g) 1152 cm^−1^ (δ (C–OH) motions coupled to δ (C=C–H) in the enolic group of the IRC); (h) 962 cm^−1^ (ν (C–OH) of enol group in the IRC coupled to δ (C–OH)) [44].

**Figure 3:**
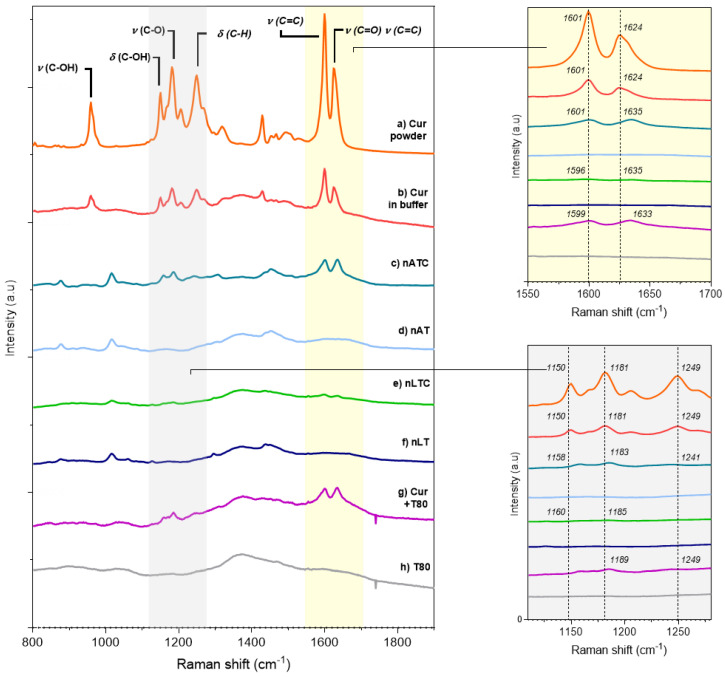
Raman spectra from (**a**) solid CUR; (**b**) CUR in Tris buffer; CUR in nanovesicles 200 µg CUR/mL (**c**) nATC and (**e**) nLTC; void nanovesicles (**d**) nAT and (**f**) nLT; (**g**) CUR in Tween 80 micelles and (**h**) Tween 80 micelles.

The spectra a) and b) in Figure 3, show the three zones of the CUR molecule affected by the interaction with Tween 80-containing lipid bilayer of nATC: the signals from IRC were shifted to higher frequencies, from 1626 to 1635 cm^−1^, and from 1150 cm^−1^ to 1158; the one from AR at 1601 cm^−1^ decreased its intensity, while those from AR at 1431 cm^−1^ and of IRC at 962 cm^−1^ disappeared. The shifting to higher frequencies has been ascribed to a reduction of conjugation of the IRC, because of displacement of the keto-enol tautomerism towards the keto form. The same changes were observed for CUR-Tween 80 and in nLTC. The decreased intensity at 1601 cm^−1^ suggests, according to [46], a reduction in the movement freedom of the aromatic rings by the establishment of van der Waals interactions of AR, either with the monooleate chains of Tween 80 in Tween 80 micelles, or with Tween 80 and lipid from nanovesicles bilayers. In addition, besides of shifted to higher frequency, the intensities of IRC 1635 cm^−1^ and of the enol peak 1158 cm^−1^ were reduced and broadened. The reduced 963/1630 ratio, which is a spectroscopic marker for the displacement of the keto-enol tautomerism towards the keto form, was null for CUR-Tween 80, nLTC and nATC, because of the disappearance of the peak at 962 cm^−1^ in these formulations [47]. This suggested the association of CUR with Tween80 containing lipid bilayers, stabilised its diketo isomer [41]. Remarkably, the signals from CUR-Tween 80 and nATC were identical, but in the more ordered matrix of nLTC bilayer, most of the peaks almost disappeared, suggesting a massive mechanical immobilization of CUR [48]. Together, the Tween 80-containing bilayers seemed to reduce the vibrational freedom of IRC, enols, and aromatic rings from CUR, and at the same time to stabilize its diketo form.

**Figure 4:**
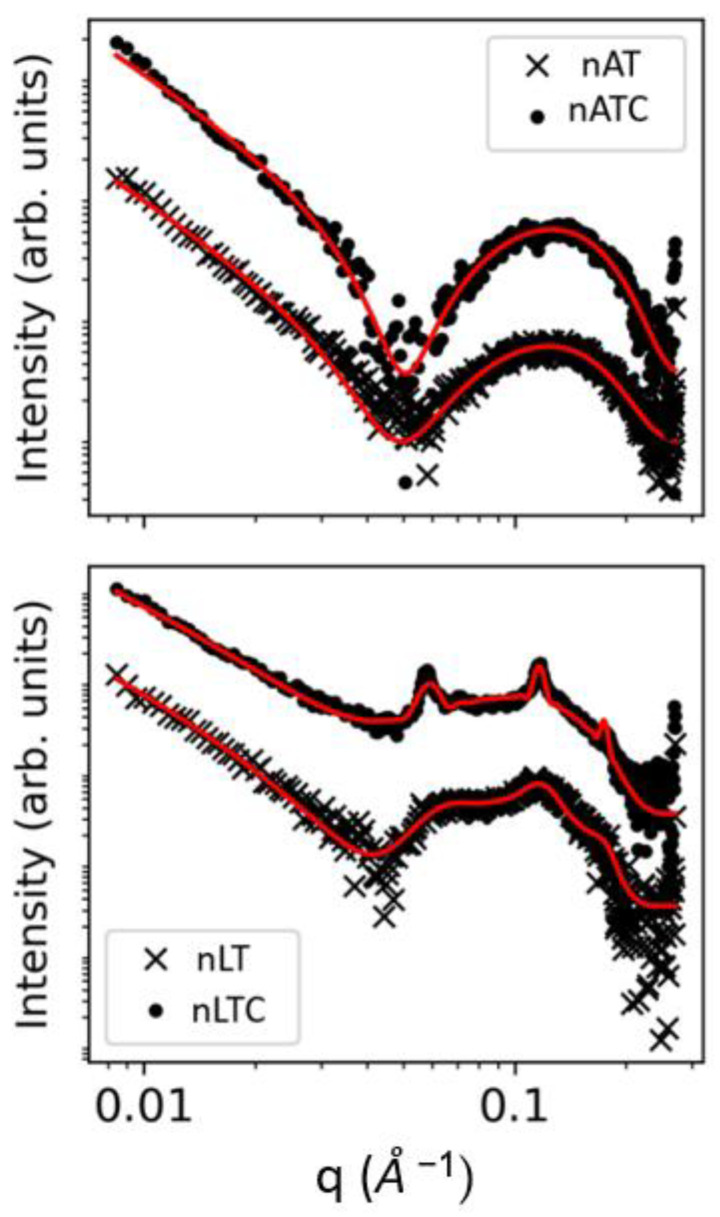
SAXS patterns in loglog scale for nAT, nATC, nLT, and nLTC systems.

SAXS: SAXS patterns showed lamellar structures in all nanovesicles (Figure 4). The presence of CUR did not alter the bilayer thickness neither of nATC not nLTC (see Table S1). In coincidence with the observed images from Cryo-EM, the structural features of nAT were compatible with unilamellar nanovesicles. The structural parameters of nATC on the other hand, were not affected by the presence of CUR, excluding minor changes in the electron density of the non-polar region, probably because of interaction with AR from CUR. In comparison, ca. 70% nLT nanovesicles were multilamellar with an average of 2.19 bilayers (the polydispersity in the number of bilayers were not taken into account). Opposing to nATC, the presence of CUR in nLTC did not affect the non-polar region, indicating that CUR was not trapped by the inner part of the bilayer. Compared to nLT, nLTC increased the number of bilayers to 6.35, while decreased the percentage of unilamellar to ca. 13%; this signified that combined with phospholipids, CUR promoted the formation of larger and ordered supramolecular nanovesicles.

Laurdan: The order and fluidity of nanovesicles bilayers was determined employing the fluorescent dye Laurdan (Table 2). The low GP of nA, nAT, and nATC bilayers indicated highly disorganized archaeolipid bilayers, where the monooleate chains from Tween 80 and CUR molecules partitioned without perturbing the disorder. Opposing to the high disorder, the relatively high FA of nA and nAT, indicated bilayers of low lipid lateral mobility, typical of archaeolipids [49]. Importantly, the FA bilayers was further increased by CUR in nATC, suggesting that CUR established additional attractive interactions with archaeolipids and/or Tween 80, increasing its microviscosity.

The disappearance of Raman peaks from CUR in nanovesicles bilayers indicated loss of vibration freedom and signified mechanical trapping of AR, enols, and IRC both in nATC and nLTC. The increased FA from nATC, besides of mechanical trapping, suggest the establishment of sticky interactions between CUR and the methyl groups perpendicular to the longitudinal carbon chains of archaeolipids, as already described for bilayers made of archaeolipids, phospholipids, and sodium cholate [50]. Such additional interactions were absent in nLTC, where CUR did not increase its FA but slightly reduced its GP. The SAXS pattern showed that in nATC, CUR altered the non-polar region of the archaeolipid bilayer, confirming that, different from nLTC, the disordered and low rotational mobility of archaeolipids favoured the deep penetration and the trapping of CUR in the bilayer.

**Figure 5:**
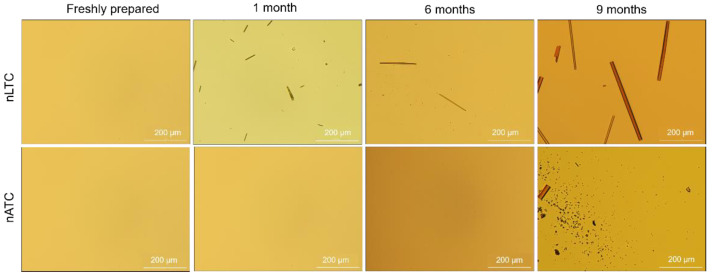
Clear field microscopy representative images showing freshly prepared and after stored at 4 °C CUR nanovesicles.

CUR release from nanovesicles: the presence of CUR crystals was screened by optical micrography of nATC and nLTC 20 µg CUR/mL suspended in 1/10 culture media at 4 °C. No crystals were observed during the first week (Figure 5). After the first month, long CUR microcrystals, between 2 µm width up to 100 µm length, that grew up to 17 µm × 350 µm after 9 months, appaired in the suspension media of nLTC, confirming the association between CUR and nLTC was less stable than between CUR and nATC. In the nATC suspension square microcristales of nearly 8 µm side and 74 µm × 18 µm rods were observed only after 9 months. Both types of nanovesicles maintained their mean size; nATC did not modify its pdi, but the pdi of nLTC was increased probably because of the big sized crystals (Figure S4).

Nanovesicles stability to nebulization: size, pdi, and CUR and lipid content of freshly prepared or stored by 6 months nATC and nLTC suspensions, were measured before and after nebulization.

**Figure 6:**
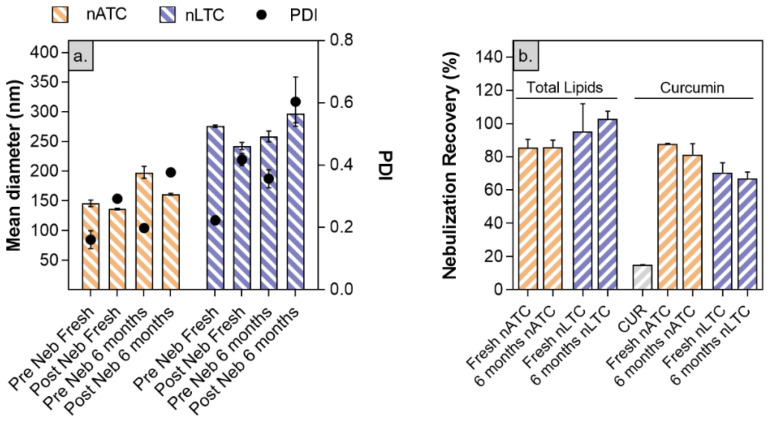
Stability of freshly prepared or stored by 6 months CUR nanovesicles to nebulization stress. (**a**) size, pdi and Z potential pre and post nebulization; (**b**) percentage of recovery of lipids and CUR post nebulization.

The mean size of freshly prepared nTLC slightly decreased from 275 ± 2 nm to 242 ± 6 nm and increased its pdi from 0.2 to 0.42 upon nebulization. The lipids recovery was 95 ± 15% and the CUR recovery was 70 ± 6%: in other words, nearly 30% CUR was lost during nebulization (big sized crystals remained trapped within the nebulizer). The mean size of freshly prepared nATC, decreased from 198 ± 10 to 161 ± 2 nm while its pdi increased from 0.20 to 0.3; the lipid recovery was 85 ± 5% and CUR 88 ± 1%, suggesting that nebulized nATC retained the CUR content. Nearly 15 ± 1% free CUR was recovered upon nebulization. After 6 months of storage, the pdi of nebulized nLTC increased to 0.61, with no changes in lipid and CUR recovery, indicating that storage did not induce additional CUR release, that nebulization stress revealed the loss of ~30% CUR (Figure 6). Instead, no changes in size and total lipids or CUR recovery were observed after 6 months stored nATC nebulization.

**Figure 7:**
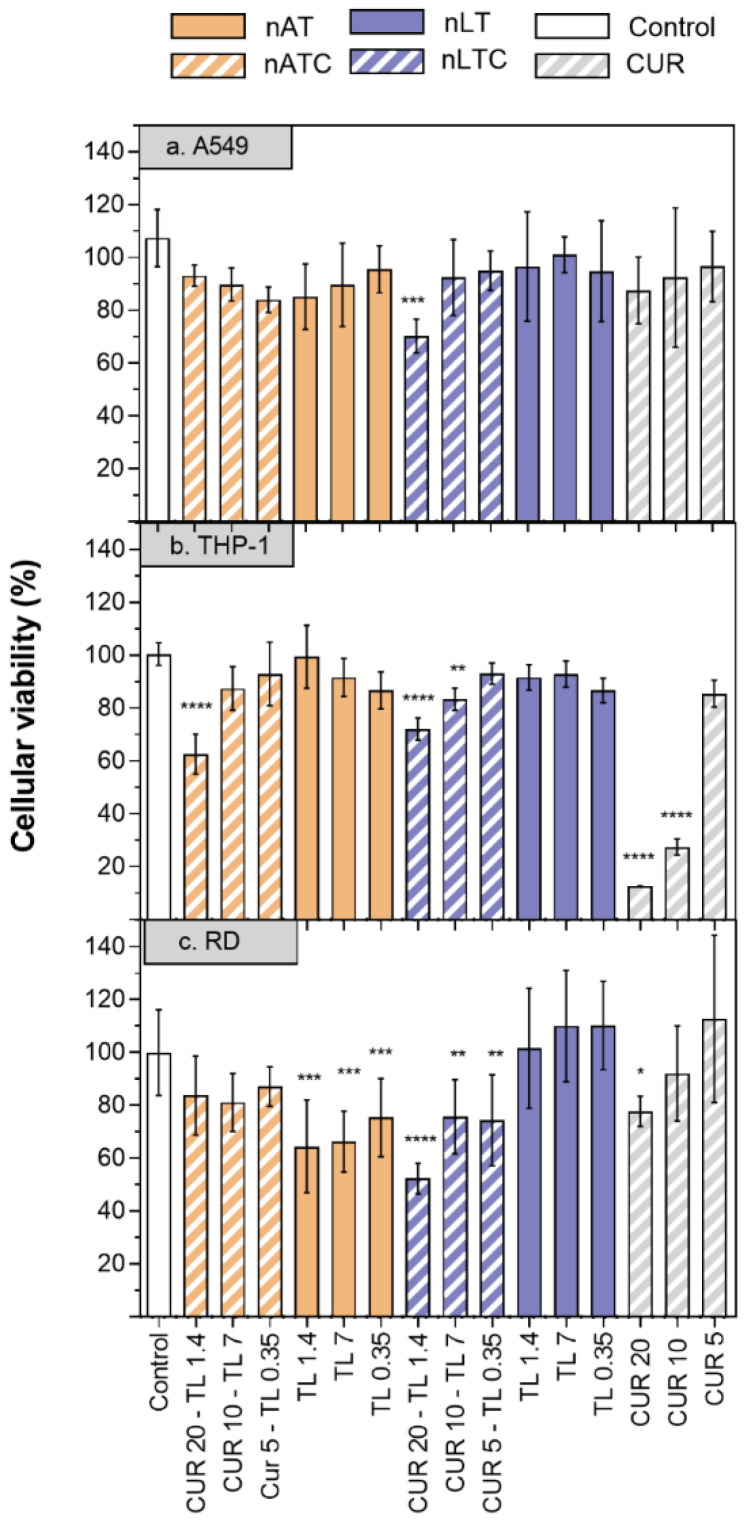
Cell viability of CUR, nATC, nAT, nLTC, and nLT in A549, THP-1 and RD cell lines. (1) CUR 20 µg/mL—total lipids 1.4 mg/mL, (2) CUR 10 µg/mL—total lipids 0.7 mg/mL, (3) CUR 5 µg/mL—total lipids 0.35 mg/mL. Values were analysed using a one-way ANOVA compared with control, * *p* < 0.05; ** *p* < 0.01; *** *p* < 0.001, **** *p* < 0.0001.

**Figure 8:**
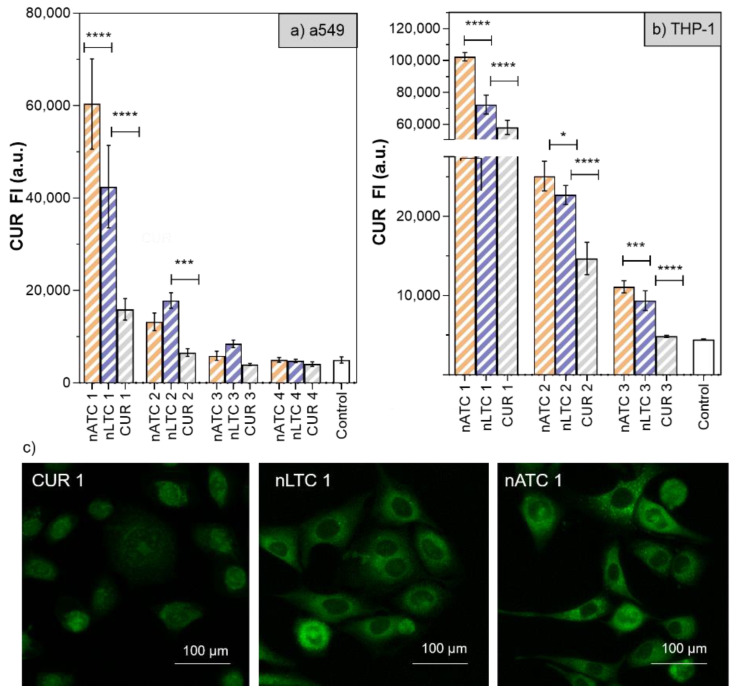
CUR internalization after 24 h incubation of free CUR and CUR nanovesicles on (**a**) A549 and (**b**) THP-1 cells. (1) CUR 20 µg/mL—total lipids 1.4 mg/mL, (2) CUR 10 µg/mL—total lipids 0.7 mg/mL, (3) CUR 5 µg/mL—total lipids 0.35 mg/mL. Values were analysed using a one-way ANOVA, * *p* < 0.05; *** *p* < 0.001, **** *p* < 0.0001). (**c**) representative images of fluorescence microscopy of intracellular CUR on A549 cells.

Cytotoxicity of CUR-nanovesicles: The cytotoxicity of CUR nanovesicles was determined by MTT in the following cell lines: A549, as a model of alveolar epithelia, THP-1 as a low SRA1 expressing macrophage model and RD as muscle cell model (Figure 7). After 24 h, 20 µg CUR/mL, and 1,4 mg lipids/mL nATC decreased by ~40% the cell viability only of THP-1. Surprisingly, a higher cytotoxicity on the three cell lines was caused by 20 µg CUR/mL nLTC, that decreased by ~30% the viability of A549 cells, by ~25% the viability of THP-1 cells and by ~50% that of RD cells. Since nATC and nLTC were relatively cytotoxic to THP-1, the release of LDH by THP-1 was included to assess for potential differences in damages caused by nATC and nLTC. Surprisingly, it was found that not only nLTC but also free CUR released LDH (Figure S6). Such release would not be owed to the endocytic uptake of CUR, since it was observed that nATC provided a higher intracellular delivery of CUR than nLTC and free CUR (Figure 8a). Potentially thus, the release of LDH would be owed to the presence of CUR crystals, observed both in nLTC and CUR suspensions, but absent in nATC.

In Figure 8a,c selection of fluorescence microscopies of A549 cells after 24 h incubation is shown.

**Figure 9:**
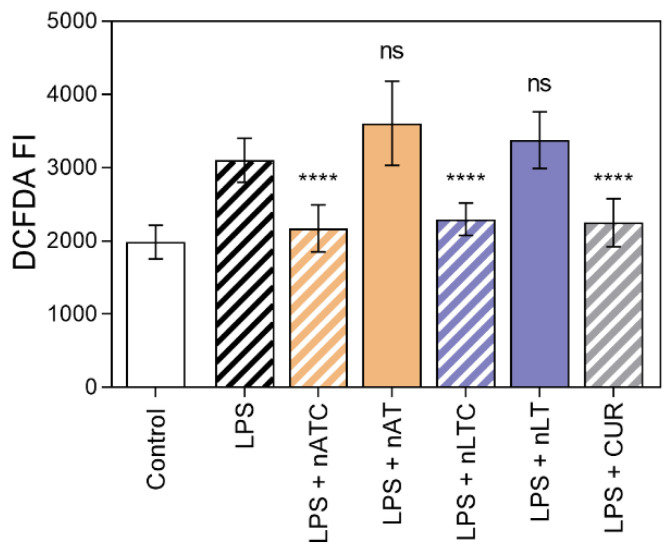
Inhibition of ROS on THP-1cells activated with LPS by nATC, nLTC, or CUR at 5 µg CUR/mL. Values were analysed using a one-way ANOVA compared with cells activated with LPS, **** *p* < 0.0001.

Effect of CUR-nanovesicles on ROS production: The production of ROS as a measure of antioxidant activity of CUR nanovesicles, was measured on LPS activated THP-1 cells. ROS was decreased to comparable levels by nLTC, nATC, and free CUR, while void nanovesicles had no antioxidant activity (Figure 9).

**Figure 10:**
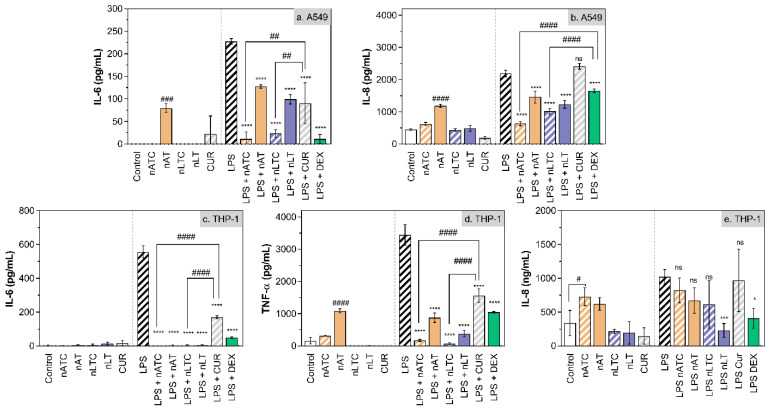
Release of pro-inflammatory cytokines by (**a**,**b**) A549 and (**c**–**e**) THP-1 cells upon incubation with 5 µg CUR/mL or 10 µg DEX/mL. Values were analysed using a one-way ANOVA compared with LPS control, * *p* < 0.05; *** *p* < 0.001, **** *p* < 0.0001. Significant differences between treatments were analyzed using a one-way ANOVA, # *p* < 0.05; ## *p* < 0.01; ### *p* < 0.001, #### *p* < 0.0001.

Effect of CUR-nanovesicles on cytokines release: Figure 10 is shown the anti-inflammatory activity of CUR nanovesicles, determined by measuring the release of IL-8, IL-6, and TNF-α by LPS activated A549 and THP1 cells. On A549 cells, nATC and nLTC reduced IL-6 to the same extent than DEX; and nATC reduced IL-8 in higher extent than nLTC and DEX. On LPS activated THP-1 cells, nATC and nLTC reduced IL-6 and TNF-α in higher extent than DEX, but without significant reduction of IL-8.

Effect of CUR-nanovesicles on the integrity of an ALI A549 cell monolayer:

The use of submerged cell cultures as a model of respiratory alveolar epithelium has several limitations arising from differences with the natural barrier. The first is related to the artificial liquid layer environment, since the natural alveolar epithelium receive nutrients only from their basal pole, while the apical pole is exposed to air [51]. The second, that in submerged cultures nanoparticles are deposited on cells driven by Brownian diffusion and agglomeration, whereas in the alveolar epithelium, nanoparticles interact with lung surfactant and then they are displaced by wetting forces to the aqueous hypophase where interact with the underlaying cells [52]. The third, is related to the expression and production of biomolecules by cells, which differs according to the environment. For example, exposure of A549 cells—cell line derived from type II epithelial alveolar cells—to an ALI is necessary to induce pulmonary surfactant secretion [53]. Furthermore, ALI cultured A549 cells increase the expression of mucin 5B (MUC5B) and alveolar epithelial type I cell marker proteins AQP5 and TTF-1 compared to submerged cultures, suggesting possible differentiation into alveolar epithelial type I cells under ALI conditions [54].

Micro-injuries to the alveolar epithelium can result in abnormal wound healing response, and potentially, to lung fibrosis [55]. The cuboidal alveolar epithelial cells type II that produce pulmonary surfactant proteins, are critically involved in the return to homeostasis, orchestrating the resolution of inflammation and initiating tissue repair [2,27,56,57].

Here, the integrity of ALI cultured A549 cells, was determined upon nebulization of CUR nanovesicles. The monolayer was also used as a model of inflamed epithelia on which the anti-inflammatory activity of CUR nanovesicles was tested. The TEER measurement is a sensitive and reliable tool to assess integrity and permeability of a cell monolayer, reflecting the paracellular ionic conductance across the cell monolayer. The transepithelial passage of neutral molecules such as LY, is related to the flux of paracellular water, and to the pore sizes of tight junctions [58].

**Figure 11:**
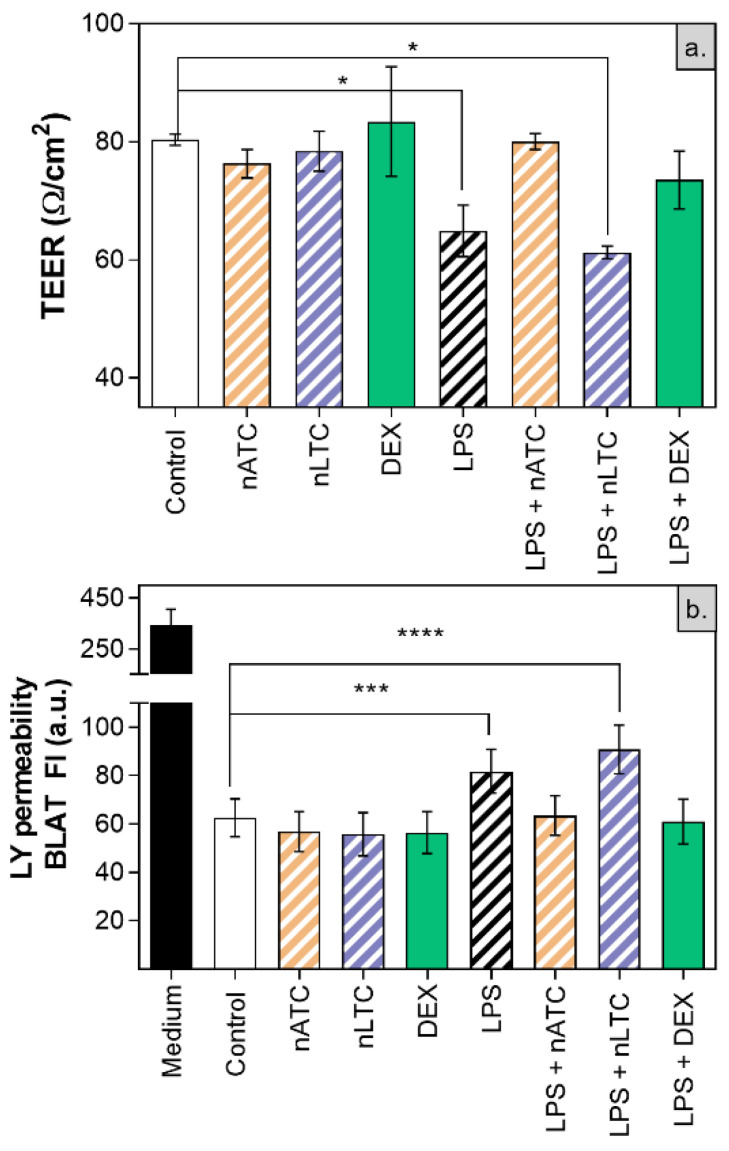
(**a**) TEER and (**b**) LY permeability after CUR nanovesicles were nebulized on the liquid-air interface of an A549 cells monolayer as an in vitro model of pulmonary alveoli. Values were analysed using a one-way ANOVA compared with control, * *p* < 0.05; *** *p* < 0.001, **** *p* < 0.0001.

**Figure 12:**
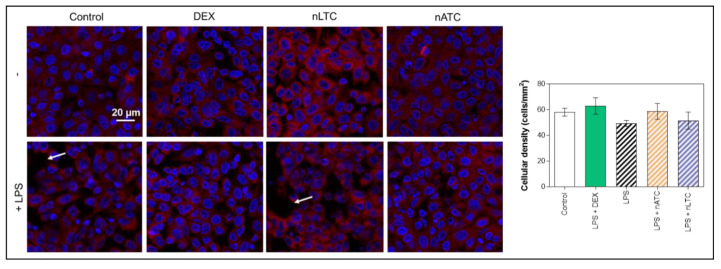
Cellular density after CUR nanovesicles were nebulized on the liquid-air interface of an A549 cells monolayer as an in vitro model of pulmonary alveoli. White arrows point out regions of the monolayers without cells.

**Figure 13:**
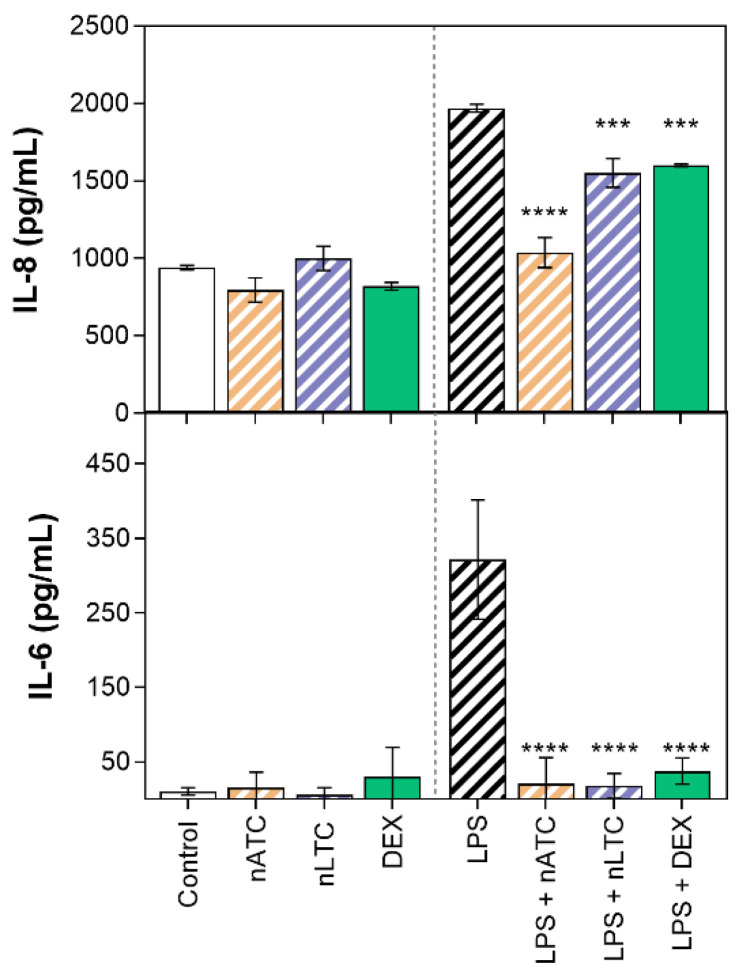
Release of pro-inflammatory cytokines from A549 ALI upon CUR nanovesicles nebulization. Values were analysed using a one-way ANOVA compared with cells activated with LPS, *** *p* < 0.001; **** *p* < 0.0001.

It was first found that none of the nebulized nanovesicles decreased the TEER value (~80 Ω/cm^2^), increased the passage of LY (Figure 11) or decreased cell density observed by fluorescence microscopy (Figure 12). Secondly, the induction of LPS activation decreased TEER to ~65 Ω/cm^2^, increasing by 20% the LY permeability and decreasing the density of the cell monolayer. Upon nLTC nebulization, the TEER value remained at ~61 Ω/cm^2^, the passage of LY increased to 30%, and the cell density remained unchanged. On the contrary, upon nATC nebulization, the inflammatory parameters were reversed: TEER was raised to ~80 Ω/cm^2^, while the LY passage and monolayer density returned to the control values; the same occurred upon DEX administration. Cytokines were determined in the basolateral compartment, as a measure of anti-inflammatory activity of nebulized nanovesicles. Remarkably, nebulized CUR-nanovesicles and DEX completely inhibited the release of IL-6; instead, while neither nLTC nor DEX affected the release of IL-8, nATC partly reduced the level of IL-8 (Figure 13).

## 4. Discussion

Inhalation is a direct pathway to the upper and lower respiratory tract, and CUR has been reported to display antiretroviral, anti-inflammatory and antifibrotic activity [59,60]. However, the clinical use of CUR is precluded by its poor solubility in aqueous media, poor stability, and low oral bioavailability [61]. The same reasons explain why, up to now, few works have addressed the challenge of delivering CUR to the lung epithelia. CUR is almost insoluble (~0.6 µg/mL) in water at pH 7, partially soluble in alkaline water, sparingly soluble in hydrocarbon solvents and readily soluble in polar solvents like DMSO, methanol [62]. Curcumin is primarily available in capsule form from commercial manufacturers [63] and the same as its oral absorption, the inhalation of free CUR is hampered because of its poor hydrophilicity. To overcome this, CUR has been derivatized it into a hydrophilic, nebulizable formulation [64] or it was loaded in liposomes [65] or polymeric and mesoporous microparticles [60,61,62,63] to be inhaled as dry powders. However, a nebulized CUR formulation is of interest, mainly because nebulizers are easy to use in infants, the elderly, patients with severely compromised lungs, and even ventilated patients. In addition, nebulization is the aerosolization mechanism best suited to inhale liposomes [64]. Liposomes, however, have previously shown not to be well suited to retain CUR, which in biological media, tends to be easily released [65].

Recently, the efficacy of nebulized CUR on an inflamed A549 ALI monolayer model was reported, tough CUR was not conveniently formulated in particulate material [64].

In here it was found that the interesting structural properties of archaeolipid bilayers alone, were not sufficient to entrap drugs, and that the addition of Tween 80 was essential to increase the CUR/lipid ratio. The Raman spectra showed that with the aid of Tween 80, a mixture of planar and angular CUR molecules was trapped into the lipid bilayers. The presence of CUR induced a sharp decrease in the fluidity of nATC bilayers, indicating sticky interactions between CUR and the Tween 80/archaeolipids bilayer. No changes in fluidity were observed in nLTC, suggesting the sticky interactions existed only between CUR and archaeolipids, being the association between CUR and Tween 80/ordinary phospholipids thus, weaker than in nATC. These findings were supported by the SAXS patterns, that showed a deep CUR penetration only within nATC bilayer. The release of CUR as a function of time, and upon nebulization confirmed that, despite of sharing comparable CUR/lipid ratio, CUR was more firmly retained into nATC than in nLTC: long micrometer-sized CUR crystal rods (nearly 30% CUR content) were immediately released from nLTC upon dilution, and a similar proportion was lost from nLTC, presumably as clogged crystals, upon nebulization. No significant variations in size, z potential and pdi were registered post nebulization.

The current paradigm indicates that any alveolar type 2 cells injury/dysfunction serves as an early initiating event leading to fibroproliferation and progressive loss of lung function [26]. The loss of alveolar type 2 cells can limit the ability for the repair of the damaged alveolus and drive fibrosis [5]. Here we found that both nATC and nLTC were captured by liquid–liquid monolayers of A549 and THP-1 cells, nATC achieving higher extent of intracellular CUR, nearly 40–50% higher than that of free CUR upon 24 h. However, in coincidence with previous data on effect of CUR in nanoliposomes [65], while 20 ug/mL CUR nATC was innocuous, nLTC reduced A549 viability by 30% after 24 h.

Excepting the firmer association of CUR in nATC and the higher cytotoxicity of nLTC, when tested on liquid–liquid A549 cells cultures no further differences in the activity of the two formulations were revealed: both reduced ROS production by THP-1 cells in comparable extent to that of free CUR, and the release of IL-6 and TNF-α by A549 cells, this time surpassing the effect of free CUR and matching that of dexamethasone. The pharmacodynamics of CUR formulated as nATC and nLTC however, presumably differed from that of free CUR; since neither nATC nor nLTC are not pH sensitive, upon endocytosed probably CUR remained stacked within endosomes, precluding its access to cytoplasmic targets or plasma membrane. Such pathway would differ from that followed by free CUR, which in solution was found to diffuse across plasma membrane, where is primarily located, as well as in the nucleus and mitochondria [66]. The consequences of such change in pharmacodynamics deserves a deeper study. On the other hand, CUR was endocytosed only when formulated as nanoparticle (nLTC or nATC), while free CUR enters the cell by difusion. Here we observed that nLTC and nATC displayed a higher antiinflammatory activity than free CUR; thus the endocytosis of CUR seemed to be of help to increase its anti-inflammatory, but not its antioxidant activity, which was the same for nLTC, nATC and free CUR.

The main difference between nATC and nLTC was revealed when 5 µg CUR/mL were nebulized along 2 min on an inflamed ALI A549 monolayer. In such conditions, only nATC increased TEER, and reduced the passage of LY (indicating the recovery of the monolayer structure). In addition, nebulized nATC reduced the levels of IL-6, and remarkably, surpassed the effect of nebulized dexamethasone, reducing the levels of IL-8. Similar repairing activity of archaeolipids on inflamed intestinal epithelia models have been described recently [67].

This is the first report on the efficacy of CUR in nanovesicles, nebulized on an ALI model of A549 cells monolayer. Nanoarchaeosomes display several key properties that distinguish them from liposomes, making them of interest as new biomaterials to prepare colloidal nebulized formulations. For instance, nanoarchaeosomes are known to withstand the mechanical stress of nebulization that, unless added with cholesterol and synthetic and expensive rigid lipids, damages liposomal bilayers [21,68]. Nanoarchaeosomes display high colloidal stability in aqueous media, being refractory to cold chain interruption because of the lack of oxidable double bonds; avoiding the need for lyophilization to increase its shelf life, simplifying their preparation [19]. The archaeolipids structure is related to that of squalene and ramified isostearic acid (ISA), a mixture of branched (mostly methyl-branched) and straight chain isomers of C18, C16, and C14 saturated fatty acids, known to combine properties of stearic acid with fluidity and solubilizing properties unsaturated fatty acids such as the oleic acid [69]. In addition, the nanoarchaesomes bilayer displays typical high local microviscosity, because of the presence of perpendicular to the longitudinal chain axe methyl groups, cardiolipins, and glycolipids [70]. Another relevant property of archaeolipids as biomaterials for lung delivery, is their ability to reduce the surface tension of the alveolar surfactant monolayer (a fact that may favour the respiratory compression-expansion dynamics), without noticeable cytotoxicity to alveolar cells [71]. Their tensioactive attribute may help to revert the damage caused by surfactant inactivation that leads to ALI/ARDS during severe inflammation [72].

The endocytosis of archaeolipids induces effects different from those of ordinary lipids composing nanoliposomes. On THP-1 macrophages, for instance, the endocytosis of archaeolipids is suggested to induce autophagy [73]. Physiological levels of autophagy are key for a correct wound healing and epithelial repair of the alveoli [74]. The reparation of the ALI model induced by nebulized nATC thus may be related to the establishment of synergy between archaeolipids and CUR. Overall, these results suggest that nATC reunite important properties for epithelia repair upon inflammatory damage, deserving further deeper exploration, particularly in relation to its ability to reduce IL-8, a strong chemotactic factor for PMN, its specific macrophage/endothelial targeting [75], and its potential antimicrobial activity.

## Figures and Tables

**Table 1:** Structural features of void and CUR-loaded nanovesicles. Results are the media of 6 lotes. * n = 2.

Sample	CUR (mg/mL)	Total Lipids (mg/mL)	ζ Average (nm)	PDI	ζ Potential (mV)	CUR	Total Lipids Recovery (%)
						Recovery (%)	
nA		14.2 ± 2.5	180 ± 10	0.25 ± 0.03	−40.2 ± 2.0		56.8 ± 10
nAC *	0.080 ± 0.04	12.4 ± 1.6	191 ± 47	0.22 ± 0.01	−35.7 ± 3.2	8.0 ± 4	49.6 ± 6.4
nAT	-	16.5 ± 0.6	216 ± 3	0.26 ± 0.01	−18.4 ± 3.5		66 ± 2.4
nATC	0.22 ± 0.09	12.9 ± 4.0	155 ± 16	0.23 ± 0.05	−20.7 ± 3.3	22.2 ± 8.7	46.5 ± 14
nL		13.8 ± 2.7	150 ± 5	0.19 ± 0.04	−3.0 ± 1.2		55.2 ± 10
nLC *	0.059 ± 0.020	11.9 ± 2.1	553 ± 222	0.919 ± 0.066	−3.7 ± 0.5	5.9 ± 2	47.6 ± 8.4
nLT	-	16.6 ± 3.4	247 ± 18	0.16 ± 0.01	−2.0 ± 0.1		66.3 ± 12
nLTC	0.17 ± 0.04	14.7 ± 3.7	278 ± 31	0.29 ± 0.23	−2.5 ± 0.7	17.2 ± 3.5	58.7 ± 15

**Table 2:** Laurdan GP and FA.

Sample	GP	FA
nA	−0.4	0.15
nAT	−0.407 ± 0.047	0.164 ± 0.013
nATC	−0.451 ± 0.009	0.215 ± 0.024
nL	0.5	0.26
nLT	0.503 ± 0.008	0. 239 ± 0.008
nLTC	0.428 ± 0.057	0.242 ± 0.007

## Data Availability

The data presented in this study are available on request from the corresponding author.

## Supplementary Materials

The following are available online at https://www.mdpi.com/article/10.3390/pharmaceutics13091331/s1, Figure S1: Curcumin quantification, Figure S2: Curcumin chemical structure, Figure S3: Curcumin suspensions, Table S1: SAXS’ model parameters obtained from nonlinear least squares procedure, Figure S4: Nanovesicles stability upon storage, Figure S5: Nanovesicles stability upon nebulization, Figure S6: Cytotoxicity of CUR-nanovesicles.
