# Supplementary material for: Reparation of an Inflamed Air-Liquid Interface Cultured A549 Cells with Nebulized Nanocurcumin
Source: Pharmaceutics. 2021 Aug 25;13(9):1331. doi: 10.3390/pharmaceutics13091331 (PMC8466083; doi:10.3390/pharmaceutics13091331)
Supplement: Supplementary file 1 [file pharmaceutics-13-01331-s001.zip › pharmaceutics-1326782-supplementary.pdf]

pharmaceutics

MDPI

Supplementary Materials: Reparation of an Inflamed Air–Liquid Interface Cultured A549 Cells with Nebulized Nanocurcumin

Maria Julia Altube, Lilen Ivonne Caimi, Cristian Huck-Iriart, Maria Jose Morilla and Eder Lilia Romero

S1. Curcumin quantification

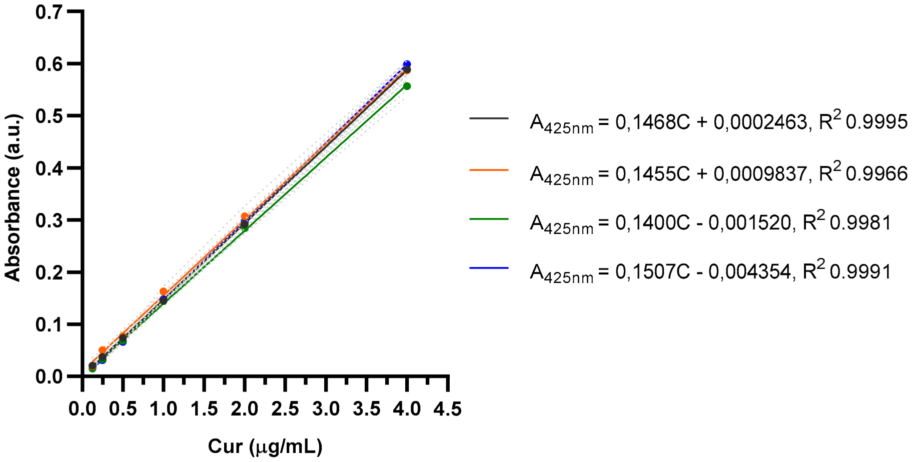

Figure S1. Standard curves of curcumin dissolved in methanol. Curves were made different days.

S2. Curcumin chemical structure

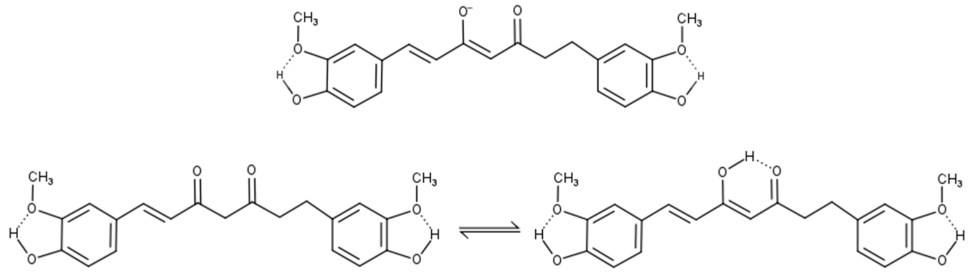

Figure S2. Chemical structure and tautomerical forms of Curcumin [40].

S3. Curcumin suspensions

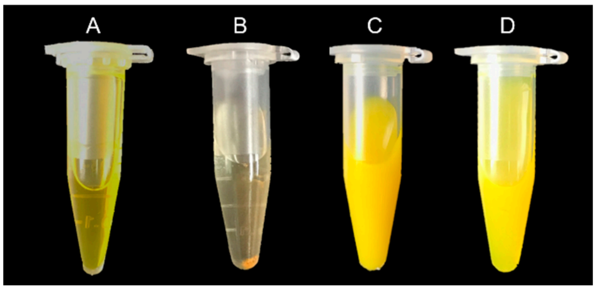

Figure S3. 200 µg/mL Cur suspensions in (**a**) methanol, (**b**) Tris buffer NaCl pH 7.4, (**c**) nATC 356 and (**d**) nLTC.

S4. SAXS model

SAXS intensity were analyzed according to the following equation

$$I(q) = S_c[w \cdot P_s + (1-w)P_m] + back(q) \quad \text{(S1)}$$

Where the scattered intensity "*I(q)*" with the weighted contribution of the scattering curve of the uni (P_s) and multilamellar structures (P_m) [31], respectively, and the background function is determined by:

$$back(q) = k_1 + \frac{k_2}{q^{\alpha}} \quad \text{(S2)}$$

Here, S_c and k_1, k_2 and$w$ are escalar and α is an exponential law for low angles contribution. The form factors for the uni and multilamellar contributions are described in the equation S3

$$P_s = 2\pi q^{-2}P_{inf}(q);\ P_m = 2\pi q^{-2}P_{inf}(q)S(q) \quad \text{(S3)}$$

Where $P_{inf}(q)$ is the infinite form factor of a bilayer structure composed by the polar head and the hydrophobic tail of the surfactant. The form factor is modelled in the following equation

$$P_{inf} = 4q^{-2}\{\Delta\rho_{CH_3}sin(qR_{CH_3}) + \Delta\rho_{-CH_2-}\{sin(q[R_{CH_3} + R_{-CH_2-}]) - sin(qR_{CH_3})\} + \Delta\rho_{head}\{sin(q[R_{CH_3} + R_{-CH_2-} + R_{head}]) - sin(q[R_{CH_3} + R_{-CH_2-}])\}\}^2 \quad \text{(S4)}$$

where $\Delta\rho_{CH3} = \rho_{CH3} - \rho_{solution}$; $\Delta\rho_{headl} = \rho_{head} - \rho_{solution}$ and $\Delta\rho_{-CH2-} = \rho_{-CH2-} - \rho_{solution}$. The lipid bilayer thickness in this model is $2(R_{head} + R_{-CH2-} + R_{CH3})$. During the fitting procedure, some of these parameters were allowed to vary within a narrow range: R_{CH3} (1.5 Å < R_{CH3} < 3.5 Å), ρ_{CH3} (0.15 e/Å^3 < ρ_{CH3} < 0.20 e/Å^3) and ρ_{-CH2-} (0.25 e/Å^3 < ρ_{-CH2-} < 0.30 e/Å^3), in accordance with data from the literature [31,33,34]. The other $P_{inf}(q)$ parameters could vary in a corresponding broader range. For the multilamellar vesicles (MLV) the Modified Caillé Theory (MCT)[31,32] was used to calculate *S(q)* in equation S3. This model considers the bending of the membrane and the fluctuations in the space between bilayers by a statistical approach. For that, a disorder parameter is added $\eta_{Caillé}$ to the equation, that can be written as [33,34]:

$$S(q) = N + 2\sum_{n=1}^{N-1}(N-n)cos(nqd)exp(-0.5772\eta_{caille}x)(\pi n)^{-\eta x} \quad \text{(S5)}$$

Where $x = (qd/2\pi)^2$. Here *N* is the number of the stacked bilayers, *d* is the repetition distance. Finally, the parameter *w* does not represent the real proportion between uni and multilamellar structures. Thus, the invariant *Q* [34] is employed instead (equation S6) where χ_s(%) is the percentage of unilamellar structures in the system.

$$\chi_s(\%) = 100 \cdot \frac{w\int_0^{\infty} q^2P_s(q)dq}{\int_0^{\infty} q^2(wP_s(q) + [1-w]P_m(q))dq} \quad \text{(S6)}$$

Models were implemented in Python 3.8 scripting using the LMfit library [35] for non-linear least square procedures.

Table S1. SAXS' model parameters obtained from nonlinear least squares procedure.

Sample	AT	ATC	LT	LTC
w	1	1	0.87 ± 0.02	0.958 ± 0.002
χ_s (%)	100	100	32 ± 1	13 ± 1

R_{CH3} (nm)	0.150 ± 0.003	0.150 ± 0.001	0.150 ± 0.001	0.153± 0.001
R_{-CH2-} (nm)	0.79 ± 0.09	0.79 ± 0.09	1.05 ± 0.01	1.03 ± 0.08
R_{head} (nm)	1.75 ± 0.06	0.170 ± 0.06	1.93 ± 0.05	1.86 ± 0.09
Thickness (nm)	5.3 ± 0.1	5.3 ± 0.1	6.3 ± 0.1	6.1 ± 0.1
ρ_{head} (e/Å^3)	0.42 ± 0.01	0.41 ± 0.01	0.40 ± 0.01	0.40 ± 0.02
ρ_{-CH2-}(e/Å^3)	0.23 ± 0.01	0.25 ± 0.02	0.25 ± 0.01	0.25 ± 0.03
ρ_{CH3}(e/Å^3)	0.17 ± 0.01	0.20 ± 0.01	0.15 ± 0.01	0.15 ± 0.01
N	--	--	2.19 ± 0.06	6.35 ± 0.05
d (nm)	--	--	10. 75 ± 0.04	10.77 ± 0.01
η_{Caille}	--	--	0.009± 0.001	0.009 ± 0.001

S5. Nanovesicles stability upon storage

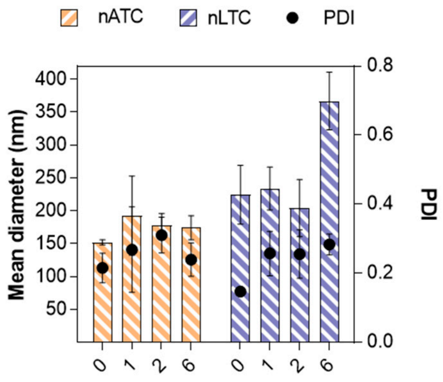

Figure S4. Stability upon storage by 1, 2 and 6 months at 4 °C of nATC and nLTC.

S6. Nanovesicles stability upon nebulization

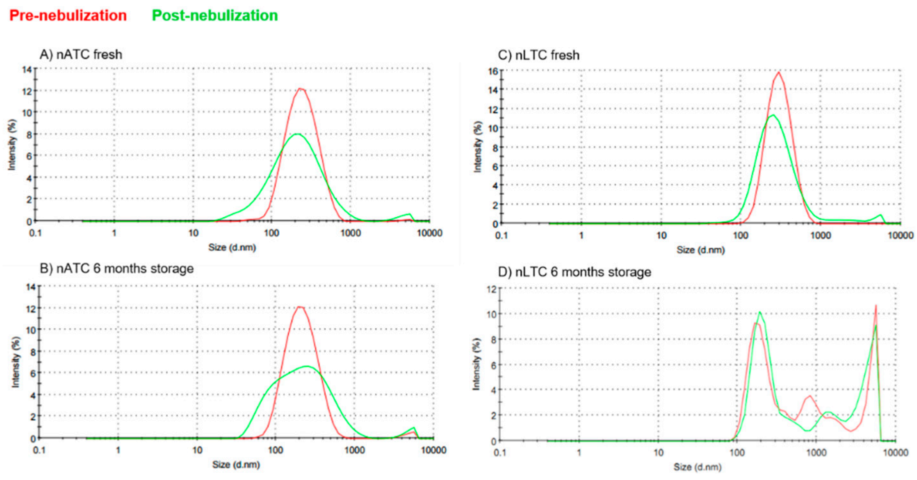

Figure S5. Changes in mean size during nebulization of a) nATC fresh, c) nLTC fresh or after 6 months incubation b) nATC and d) nLTC.

S7. Cytotoxicity of CUR-nanovesicles

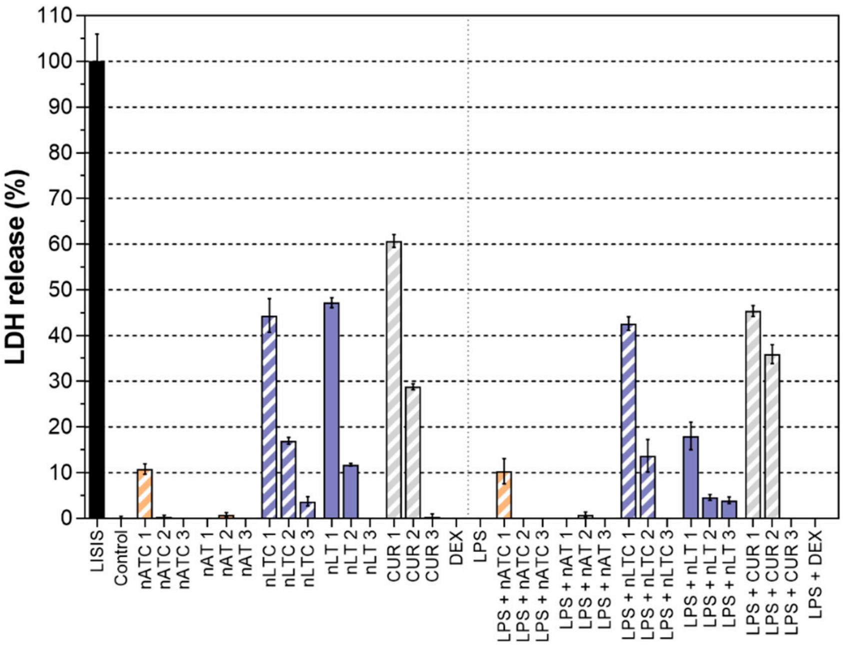

Figure S6. LDH release after 24 h incubation of free CUR and CUR loaded nanovesicules on THP-1 cells 1) CUR 20 μg/mL – total lipids 1.4 mg/mL, 2) CUR 10 μg/mL – total lipids 0.7 mg/mL, 3) CUR 5 μg/mL – total lipids 0.35 mg/mL.

References:

[30] M.J. Altube, M.M.B. Martínez, B. Malheiros, P.C. Maffía, L.R.S. Barbosa, M.J. Morilla, E.L. Romero, Fast Biofilm Penetration and Anti-PAO1 Activity of Nebulized Azithromycin in Nanoarchaeosomes, Mol. Pharm. 2020, 17. https://doi.org/10.1021/acs.molpharmaceut.9b00721.

[31] R.M. Fernandez, K.A. Riske, L.Q. Amaral, R. Itri, M.T. Lamy, Influence of salt on the structure of DMPG studied by SAXS and optical microscopy, Biochim. Biophys. Acta BBA - Biomembr. 2008, 1778. https://doi.org/10.1016/j.bbamem.2007.12.005.

[32] R. Zhang, S. Tristram-Nagle, W. Sun, R.L. Headrick, T.C. Irving, R.M. Suter, J.F. Nagle, Small-angle x-ray scattering from lipid bilayers is well described by modified Caillé theory but not by paracrystalline theory, Biophys. J. 1996, 70. https://doi.org/10.1016/S0006-3495(96)79576-0.

[33] R. Zhang, R.M. Suter, J.F. Nagle, Theory of the structure factor of lipid bilayers, Phys. Rev. E. 1994, 50. https://doi.org/10.1103/PhysRevE.50.5047.

[34] A.F. Craievich, Small-Angle X-ray Scattering by Nanostructured Materials, in: L. Klein, M. Aparicio, A. Jitianu (Eds.), Handb. Sol-Gel Sci. Technol., Springer International Publishing, Switzerland Cham, 2016. https://doi.org/10.1007/978-3-319-19454-7_37-1.

[35] M. Newville, T. Stensitzki, D.B. Allen, A. Ingargiola, LMFIT: Non-Linear Least-Square Minimization and Curve-Fitting for Python, Zenodo, 2014. https://doi.org/10.5281/ZENODO.11813. Available online: https://cars9.uchicago.edu/software/python/lmfit_MinimizerResult/ (accessed on 15 July 2021).

[40] Jagannathan, R.; Abraham, P.M.; Poddar, P. Temperature-Dependent Spectroscopic Evidences of Curcumin in Aqueous Medium: A Mechanistic Study of Its Solubility and Stability. The Journal of Physical Chemistry B 2012, 116, doi:10.1021/jp3050516.
